# Multicentre analysis of seizure outcome predicted by removal of high-frequency oscillations

**DOI:** 10.1093/brain/awae361

**Published:** 2024-11-12

**Authors:** Vasileios Dimakopoulos, Jean Gotman, Petr Klimes, Nicolas von Ellenrieder, Shi Bei Tan, Garnett Smith, Stephen V Gliske, Margarita Maltseva, Minette Krisel Manalo, Martin Pail, Milan Brazdil, Dorien van Blooijs, Maryse A van ‘t Klooster, Sarah Johnson, Samantha Laboy, Debora Ledergerber, Lukas Imbach, Christos Papadelis, Michael R Sperling, Maeike Zijlmans, Jan Cimbalnik, Julia Jacobs, William C Stacey, Birgit Frauscher, Johannes Sarnthein

**Affiliations:** Klinik für Neurochirurgie, Universitätsspital Zürich, Universität Zürich, 8091 Zurich, Switzerland; Montreal Neurological Institute & Hospital, McGill University, Montreal, Quebec H3A 2B4, Canada; Montreal Neurological Institute & Hospital, McGill University, Montreal, Quebec H3A 2B4, Canada; Montreal Neurological Institute & Hospital, McGill University, Montreal, Quebec H3A 2B4, Canada; Department of Biomedical Engineering, University of Michigan, Ann Arbor, MI 48109, USA; Department of Pediatrics, University of Michigan, Ann Arbor, MI 48109, USA; Department of Neurosurgery, University of Nebraska Medical Center, Omaha, NE 68198, USA; Alberta Children’s Hospital Research Institute, Hotchkiss Brain Institute, Cumming School of Medicine, University of Calgary, Calgary, Alberta T2N 4N1, Canada; Epilepsy Center Frankfurt Rhine-Main and Department of Neurology, Goethe-University, 60590 Frankfurt am Main, Germany; Alberta Children’s Hospital Research Institute, Hotchkiss Brain Institute, Cumming School of Medicine, University of Calgary, Calgary, Alberta T2N 4N1, Canada; Brno Epilepsy Center, 1st Department of Neurology, St. Anne´s University Hospital and Faculty of Medicine, Masaryk University, 602 00, Brno, Czech Republic, member of ERN EpiCARE; Brno Epilepsy Center, 1st Department of Neurology, St. Anne´s University Hospital and Faculty of Medicine, Masaryk University, 602 00, Brno, Czech Republic, member of ERN EpiCARE; Department of Neurology and Neurosurgery, University Medical Centre Utrecht Brain Center, Part of ERN EpiCARE, P.O. box 85500, 3508 GA Utrecht, The Netherlands; Stichting Epilepsie Instellingen Nederland (SEIN), Postbus 540, 2130 AM Hoofddorp, The Netherlands; Department of Neurology and Neurosurgery, University Medical Centre Utrecht Brain Center, Part of ERN EpiCARE, P.O. box 85500, 3508 GA Utrecht, The Netherlands; Department of Neurology, Thomas Jefferson University, Philadelphia, PA 19107, USA; Neuroscience Research, Jane and John Justin Institute for Mind Health, Cook Children’s Health Care System, Fort Worth, TX 76104, USA; Swiss Epilepsy Center, Clinic Lengg, 8008 Zurich, Switzerland; Swiss Epilepsy Center, Clinic Lengg, 8008 Zurich, Switzerland; Neuroscience Research, Jane and John Justin Institute for Mind Health, Cook Children’s Health Care System, Fort Worth, TX 76104, USA; Department of Neurology, Thomas Jefferson University, Philadelphia, PA 19107, USA; Department of Neurology and Neurosurgery, University Medical Centre Utrecht Brain Center, Part of ERN EpiCARE, P.O. box 85500, 3508 GA Utrecht, The Netherlands; Stichting Epilepsie Instellingen Nederland (SEIN), Postbus 540, 2130 AM Hoofddorp, The Netherlands; Brno Epilepsy Center, 1st Department of Neurology, St. Anne´s University Hospital and Faculty of Medicine, Masaryk University, 602 00, Brno, Czech Republic, member of ERN EpiCARE; Alberta Children’s Hospital Research Institute, Hotchkiss Brain Institute, Cumming School of Medicine, University of Calgary, Calgary, Alberta T2N 4N1, Canada; Department of Biomedical Engineering, University of Michigan, Ann Arbor, MI 48109, USA; Department of Neurology and BioInterfaces Institute, University of Michigan, Ann Arbor, MI 48109, USA; Montreal Neurological Institute & Hospital, McGill University, Montreal, Quebec H3A 2B4, Canada; Department of Neurology & Biomedical Engineering, Duke University, Durham, NC 27705, USA; Klinik für Neurochirurgie, Universitätsspital Zürich, Universität Zürich, 8091 Zurich, Switzerland

**Keywords:** ripples, fast ripples, automated detection, epilepsy surgery, intracranial EEG

## Abstract

In drug-resistant focal epilepsy, planning surgical resection can involve presurgical intracranial EEG (iEEG) recordings to detect seizures and other iEEG patterns to improve postsurgical seizure outcome. We hypothesized that resection of tissue generating interictal high-frequency oscillations (HFOs, 80–500 Hz) in the iEEG predicts surgical outcome.

In eight international epilepsy centres, iEEG was recorded during the presurgical evaluation of patients. The patients were of all ages, had epilepsy of all types, and underwent surgical resection of a single focus aiming at seizure freedom. In a prospective analysis, we applied a fully automated definition of HFO that was independent of the dataset. Using an observational cohort design that was blinded to postsurgical seizure outcome, we analysed HFO rates during non-rapid-eye-movement sleep. If channels had consistently high rates over multiple epochs, they were labelled the ‘HFO area’. After HFO analysis, centres provided the electrode contacts located in the resected volume and the seizure outcome at follow-up ≥24 months after surgery. The study was registered at www.clinicaltrials.gov (NCT05332990).

We received 160 iEEG datasets. In 146 datasets (91%), the HFO area could be defined. The patients with a completely resected HFO area were more likely to achieve seizure freedom in comparison to those without [odds ratio 2.61, 95% confidence interval (CI) 1.15–5.91, *P* = 0.02]. Among seizure-free patients, the HFO area was completely resected in 31 and not completely resected in 43. Among patients with recurrent seizures, the HFO area was completely resected in 14 and not completely resected in 58. When predicting seizure freedom, the negative predictive value of the HFO area (68%, CI 52–81) was higher than that for the resected volume as a predictor by itself (51%, CI 42–59, *P* = 4 × 10^−5^). The sensitivity and specificity for complete HFO area resection were 0.88 (CI 0.72–0.98) and 0.39 (CI 0.25–0.54), respectively, and the area under the curve was 0.83 (CI 0.58–0.97), indicating good predictive performance.

In a blinded cohort study from independent epilepsy centres, applying a previously validated algorithm for HFO marking without the need for adjusting to new datasets allowed us to validate the clinical relevance of HFOs to plan the surgical resection.

## Teaser

Can postsurgical seizure outcomes in epilepsy patients be improved? In an observational study based on prospective blinded analysis of intracranial recordings, Dimakopoulos *et al.* found that removal of the area of brain tissue generating high frequency oscillations was associated with higher rates of seizure freedom.

## Introduction

Drug-resistant focal epilepsy can be treated by surgical resection, with the goal of removing the epileptogenic zone (EZ),^1^ defined as the minimum brain area whose resection leads to freedom from seizures.^2,3^ The aim of resective epilepsy surgery is seizure freedom (labelled ILAE1),^4^ and surgeries are often guided by placement and recording of intracranial EEG (iEEG) to determine the resection volume (RV) that has the best chance of containing the EZ.^5^ Estimating the EZ typically requires recording seizures, which is challenging during the limited duration of the iEEG. There is great interest in also using the much longer interictal (i.e. between seizures) data to estimate the EZ.

Although there are several interictal markers for the EZ being discussed, we focus here on high-frequency oscillations (HFOs).^6-10^ A recent meta-analysis of 31 studies and >700 patients clearly indicated the potential of HFOs to delineate the EZ.^11^ To test HFO analysis in a rigorous reproducible way and motivated by a small study,^7^ we designed an international observational cohort study with prospective data analysis and pre-registered the study protocol,^9^ highlighted as an example for ‘ethos of rigour and reproducibility’.^12^ Eight centres contributed retrospectively collected clinical iEEG data. We then prospectively applied a fully automated definition of ‘HFO area’, which was independent of each dataset and was blinded to postsurgical seizure outcome.

We aimed to define the clinical relevance, robustness and generalizability of automated HFO analysis and how it compares with current clinical practice. We hypothesized that complete removal of the HFO area will render the patient seizure free, whereas incomplete removal will result in recurrent seizures.

## Materials and methods

### Study design

In a preparatory step of the study, eight independent epilepsy centres (Supplementary Table 1) agreed on patient inclusion criteria, planned methods of analysis, and outcome measures. The study protocol was then pre-registered.^9^ Study centres first provided the iEEG datasets that fulfilled the requirements from the study protocol.^9^ The HFO analysis was then done centrally at a single centre (Universitätsspital Zürich) by researchers (V.D. and J.S.) who were blinded with respect to clinical parameters. HFOs were defined by an automated detector described previously^7,10^ and outlined below (in the ‘Definition of the HFO area’ section). Of note, the HFO algorithm was developed on independent datasets and not optimized for this cohort of patients, providing a fully automated and objective application of the algorithm.^7,8,10^ Only after the HFO analysis was completed, we matched its results to the seizure outcome after epilepsy surgery. With this held-out data and the blinded study design, this study performed prospective analysis and simulates real clinical use. The term ‘prospective’ refers to the fact that our analysis followed a predetermined, pre-registered protocol, with HFO analysis conducted in a blinded fashion.

### Patient inclusion criteria

We included patients with drug-resistant focal epilepsy who fulfilled the following criteria^9^:

- Patients underwent iEEG recordings with subdural electrodes (electrocorticography, ECoG) and/or stereotactic depth electrodes (sEEG) as part of their presurgical evaluation.
- Patients underwent complete surgical resection of a single focus with the expectation of seizure freedom (i.e. entire focus removed). This excludes surgeries with a palliative indication and patients with multiple foci.
- Follow-up for ≥24 months for post-surgical outcome.^13^

**Figure 1:**
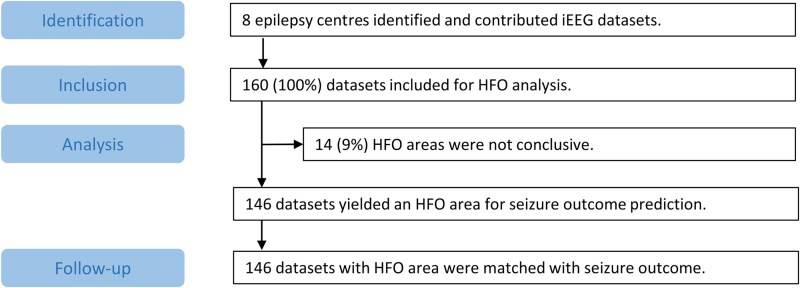
**STROBE flow chart of patient selection.** HFO = high-frequency oscillation; iEEG = intracranial EEG; STROBE = strengthening the reporting of observational studies in epidemiology.

No restrictions were applied with respect to epilepsy type, patient age or pathology. No patients used in the original studies validating this method^7,8,10^ were included. The flow chart (Fig. 1) provides information on the total group of 160 patients.

### Outcomes

The primary end point was seizure outcome with follow-up ≥24 months after surgery, as defined by the International League Against Epilepsy (ILAE) classification^4^: seizure freedom (ILAE1) or recurrent seizures (ILAE2–6).

### Bias mitigation

We used REDCap case report forms^14^ to enable transparent, reproducible and rigorous clinical research. The researchers who performed the HFO analysis were blinded to the clinical information of the patients to avoid any bias in delineating an HFO area favourable to the seizure outcome. Each study centre was asked to transfer the clinical information of the patients after the HFO analysis was completed. The study centres then documented which channels covered tissue that was removed and they reported the seizure outcome of each patient. The HFO area results were revealed to the study centres only after they had transmitted the clinical information of each patient. The centres then indicated whether a patient had a ‘completely resected HFO area’ (crHFO-area) or not. With this procedure, we ensured that the investigators from the study centres were not biased towards crHFO-area when patients achieved favourable seizure outcome. The Clinical Trials Center (CTC) Zurich supervised the study and ensured regulatory compliance and protocol adherence.

### Ethical considerations

The study was registered at www.clinicaltrials.gov (NCT05332990). The patients’ consent was obtained according to the Declaration of Helsinki. Each study centre obtained ethics approval by their local ethics committee. All data were pseudonymized before sharing. Each patient was identified by a code, and the code remained at the study centre. Each study centre signed a data transfer agreement with Universitätsspital Zürich.

### Electrode types and implantation sites

Intracranial electrodes (sEEG and/or ECoG) had been placed according to the findings of the non-invasive presurgical evaluation. Following their standard procedure, study centres used pre-implantation MRI and post-implantation MRI or CT images to locate each electrode contact and whether the contact was in the RV or not.

### Data preprocessing

We selected data that were recorded during nights while the patients were in non-REM sleep as indicated by the study centres. Each centre provided a list of non-cephalic channels, artefact-ridden channels and markings of EEG sections with artefacts that were excluded from further analysis. The sampling rates ranged from 2000 to 5000 Hz, depending on the centre, but all data were downsampled to 2000 Hz using the polyphaser anti-aliasing filter in MATLAB to ensure consistency. We applied bandpass filtering (80–500 Hz) uniformly across all datasets covering both ripple (80–240 Hz) and fast ripple (250–500 Hz) frequencies. We kept these settings consistent across all centres to minimize variability in HFO detection. The iEEG was transformed to bipolar channels made from adjacent contacts. The researchers at Universitätsspital Zürich (V.D. and J.S.) divided the data into 5 min epochs. All eligible 5 min epochs from all nights entered subsequent analysis.

### Definition of the HFO area

The detection of HFOs and the HFO area were described in detail in the study protocol.^9^ In brief, the HFO detector identified ripples (80–240 Hz) that co-occurred with fast ripples (250–490 Hz). In each recording epoch, we computed the HFO rate by dividing the HFO count per channel by the duration of the epoch in minutes. We analysed the spatial distribution of HFO rates across channels in each patient. The ensemble of the channels whose rates exceeded the rate threshold (95th percentile of the HFO rate distribution) were candidates to be included in the HFO area.

We tested whether the candidate HFO channels showed consistently high HFO rates over epochs. We quantified the temporal consistency of the HFO area over the ensemble of recording epochs by counting the percentage of epochs that each channel spent in the HFO area. The channels with ≥50% temporal consistency constituted the final HFO area. If the temporal consistency remained <50% for all channels, we considered the HFO area an inconclusive measure and we did not make a prediction on that particular patient.^7,9,10,15^Supplementary Fig. 1 illustrates how we determined the HFO area in an example patient. Given that the automated HFO algorithm has been proved to detect clinically relevant HFOs efficiently and to discard transient artefacts, there was no manual rejection of events in this fully automated algorithm.^7-10^ The code for the detector is available at https://github.com/ZurichNCH/Automatic-High-Frequency-Oscillation-Detector.

### Using the HFO area to predict postsurgical seizure outcome

The primary outcome was seizure freedom (ILAE1). We hypothesized that the HFO area delineates the epileptogenic tissue. Therefore, we assumed that complete removal of this HFO area would render the patient seizure free, whereas incomplete removal would lead to recurrent seizures. If it was not removed, the patient should continue to have seizures. Thus, as the elements of the confusion matrix,^11^ we defined as true positive (TP) a patient in whom the HFO area was not fully located within the RV (non-crHFO-area; non-complete, i.e. incomplete resection of the HFO area), where at least one channel of the HFO area was not resected and the patient suffered from recurrent seizures (ILAE2–6). We defined as true negative (TN) a patient in whom the HFO area was fully located inside the RV (crHFO-area; complete resection of the HFO area) and who became seizure free. We defined as false positive (FP) a patient in whom the HFO area was not fully located inside the RV but who achieved seizure freedom (ILAE1). We defined as false negative (FN) a patient in whom the HFO area was fully located within the RV but who suffered from recurrent seizures.

### Postsurgical seizure outcome in current clinical practice

The patients included in this study had resections guided by the clinical identification of the seizure focus, and all were expected to achieve seizure freedom because the entire focus was within the resected volume. None of the participating study centres used HFO detection routinely as part of their surgical work-up. Similar to the above statistical approach, we defined as TN a patient in whom the resection resulted in postsurgical seizure freedom. We defined as FN a patient who suffered from recurrent seizures. Given that the inclusion criteria required that all seizure focus electrodes were resected, we conclude that TP = 0 and FP = 0.

### Statistical analysis

We calculated the positive predictive value (PPV) as PPV = TP / (TP + FP), the negative predictive value (NPV) as NPV = TN / (TN + FN), sensitivity = TP / (TP + FN), specificity = TN / (TN + FP), and accuracy = (TP + TN) / *n*. We considered that HFO and RV were two diagnostic tests and that we could determine their negative predictive values NPV(HFO) and NPV(RV). Given that for RV we concluded that TP = 0 and FP = 0, we cannot calculate PPV, sensitivity or specificity.

We compared NPV(HFO) and NPV(RV) as follows. Given that both of the tests were applied on the same cohort of patients, we conducted a paired analysis in R.^16^ We organized our data into a paired format with the ‘tab.paired()’ function from the DTComPair package.^17^ We used the ‘pv.rpv()’ function^18^ on our paired dataset to evaluate any significant difference between NPV(HFO) and NPV(RV). This method allows calculation of the relative deviation in NPV. We tested the null hypothesis of whether the ratio of the NPV values [NPV(HFO) / NPV(RV)] is equal to one (i.e. they do not deviate). The analysis yielded a relative performance measure, a confidence interval (CI) and a *P*-value to determine the significance of the deviation in NPV between the two diagnostic tests.

When studying the relationship between crHFO-area and ILAE1 outcome, we followed the statistical approach of the meta-analysis.^11^ We computed a pooled proportion along with Clopper–Pearson 95% CIs.^19^ In a contingency analysis, we presented outcomes as odds ratios (ORs) with 95% CIs based on the binomial distribution. In the summary receiver operating characteristic (SROC) curve, we calculated the pooled sensitivity, specificity and area under the curve (AUC).^20^ The AUC value was defined between 0.5 and 0.75 as a weak predictive performance and between 0.76 and 0.92 as a good predictive performance.^20,21^

In an exploratory *post hoc* analysis, we evaluated whether specific data variables influenced the seizure outcome prediction above chance level using a linear fixed effects model. We set our response variable, *y*, as the seizure outcome. We assigned to *y* the binary value ‘1’ for TP or TN, and ‘0’ otherwise. As fixed effects, we modelled age, sex, pathology, electrode type, number of recording channels (*i*), number of channels in the HFO area (*j*), number of resected channels (*k*), crHFO-area/non-crHFO-area, and follow-up duration (fu). We performed the quantization of the effects by dividing into ranges that corresponded to the modes in the data distribution. The formula of our linear fixed effects model was:


y∼age+sex+pathology+elecType+i+j+k+crHFOarea+fu

We corrected for multiple comparisons using the false discovery rate method.

## Results

### Patient characteristics

Between 14 July 2022 and 9 January 2024, we received 160 iEEG datasets from eight study centres for HFO analysis (Fig. 1 and Supplementary Table 1). All patients underwent epilepsy surgery before 2022. We report the range of years for the group, not identifying year for the individual, because ‘year’ has been deemed to be personally identifying information in this rare dataset. Only individuals with a single seizure focus resection were included, excluding patients with more than one focus. The vast majority of patients with mesial temporal lobe epilepsy had mesial temporal sclerosis (27 of 33 = 82%). In 14 datasets (9% of 160 datasets, six female (47%), median age 23 years, number of non-REM epochs range 12–12, median follow-up duration 38 months, range 24–115 months, we did not find a conclusive HFO area. These patients were excluded from subsequent analysis. The HFO analysis was conclusive in 146 datasets [91% of 160 datasets, 78 female (53%), median age 29 years, number of non-REM epochs range 12–24, median follow-up duration 39 months, range 24–280 months; Supplementary Table 1]. In the final group of 146 patients, we analysed the HFO area with respect to the postsurgical seizure outcome.

### Electrode numbers

Among the 160 patients, 156 had depth electrodes (sEEG), four had only subdural electrodes (ECoG), and 11 had both types of electrodes. They had a total of 13 787 bipolar electrode channels (median 78 per patient), of which were 2611 resected electrode channels (median 11 per patient). In the 146 patients with conclusive HFO area, 387 channels were in the HFO area (median of two channels per patient, range, 1–7). This small number of channels reflects the parameters of the algorithm that were chosen to select a small HFO area.

### Prediction of postsurgical seizure outcome

For each of the 146 patients, we evaluated whether the HFO area was completely resected (crHFO-area) or not completely resected (non-crHFO-area**)**. Among seizure-free patients (ILAE1), the HFO area was completely resected in 31 patients (21%, TN) and not completely resected in 43 patients (29%, FP). Among patients with recurrent seizures (ILAE2–6), the HFO area was completely resected in 14 patients (10%, FN) and not completely resected in 58 patients (40%, TP). From these values we obtained NPV(HFO) = 68%, CI 52–81.

**Figure 2:**
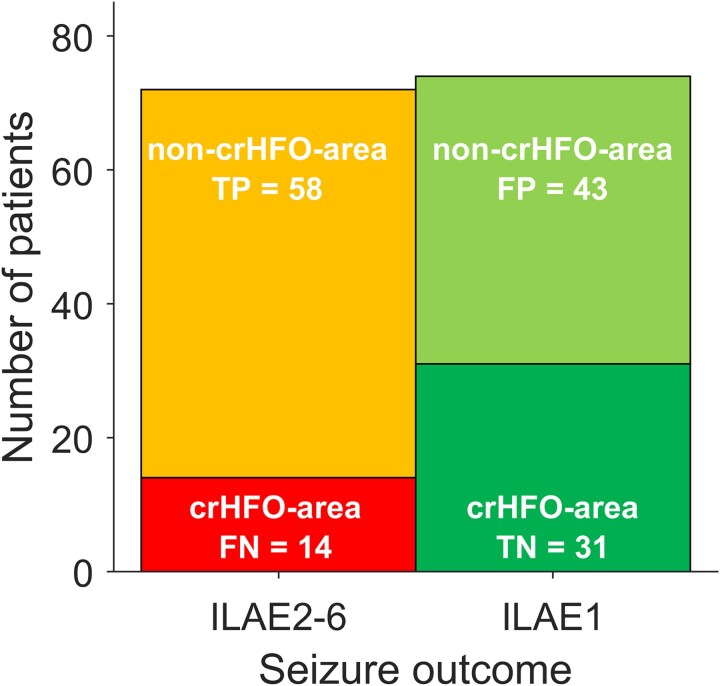
**Complete resection of HFO area compared with postsurgical seizure outcome.** There were 74 of 146 (51%) patients who achieved seizure freedom in clinical practice (ILAE1), in 31 of whom the HFO area was completely resected (crHFO-area, dark green field). There were 72 patients without seizure freedom (ILAE2–6), in 14 of whom the HFO-area was completely resected (red field). crHFO-area = completely resected HFO area; HFO = high-frequency oscillation; ILAE = International League Against Epilepsy.

The aim of our study was to compare the HFO analysis with current clinical practice, in which all relevant information, including the seizure focus identified by iEEG, is combined to decide the extent of the RV. By inclusion criteria, the focus was removed in all these patients. Among the 146 patients, 74 were seizure free (ILAE1; Fig. 2), yielding NPV(RV) = 51%. CI 42–59, i.e. the seizure-free rate. The 14 patients without conclusive HFO area had similar NPV(RV) (6 of 14 = 43% seizure free). Given that for RV, TP = 0 and FP = 0, we cannot calculate PPV, sensitivity or specificity. The NPV(HFO) (68%, CI 52–81) was significantly higher than NPV(RV) (51%, CI 42–59) *P* = 4 × 10^−5^). The relative deviation between NPV(HFO) and NPV(RV) was 1.34, CI 1.13–1.62, standard log error = 0.009.

### The pooled ILAE1 proportion with crHFO-area

**Figure 3:**
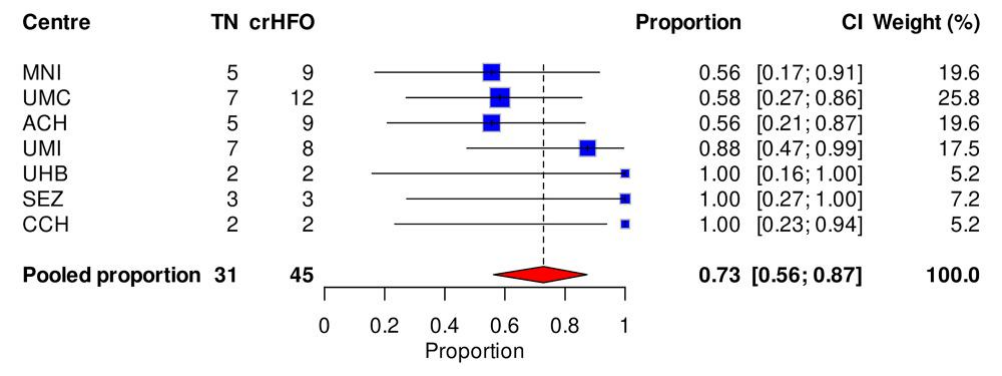
**The pooled ILAE1 proportion with crHFO-area.** Each study centre is represented by a blue square; the size of the square represents the relative statistical weight of each study centre, with larger squares indicating study centres that carry greater statistical weight in the overall results. The pooled data with 95% confidence interval (CI) is shown by a red diamond. Note that centre JUH (Jefferson University Hospital) does not appear in this analysis because it had no patients with crHFO-area. crHFO-area = completely resected high-frequency oscillation area.

There were 45 patients who underwent complete HFO-area resection, and of these, 31 achieved ILAE1 (Fig. 3). The overall pooled ILAE1 proportion of patients with crHFO-area was 73%, CI 56–88 (*P* = 2 × 10^−20^; Fig. 3). There was a heterogeneity of 4.3% (*I*^2^ = 4.3%, *P* = 0.38) for the ILAE1 patient group.

**Figure 4:**
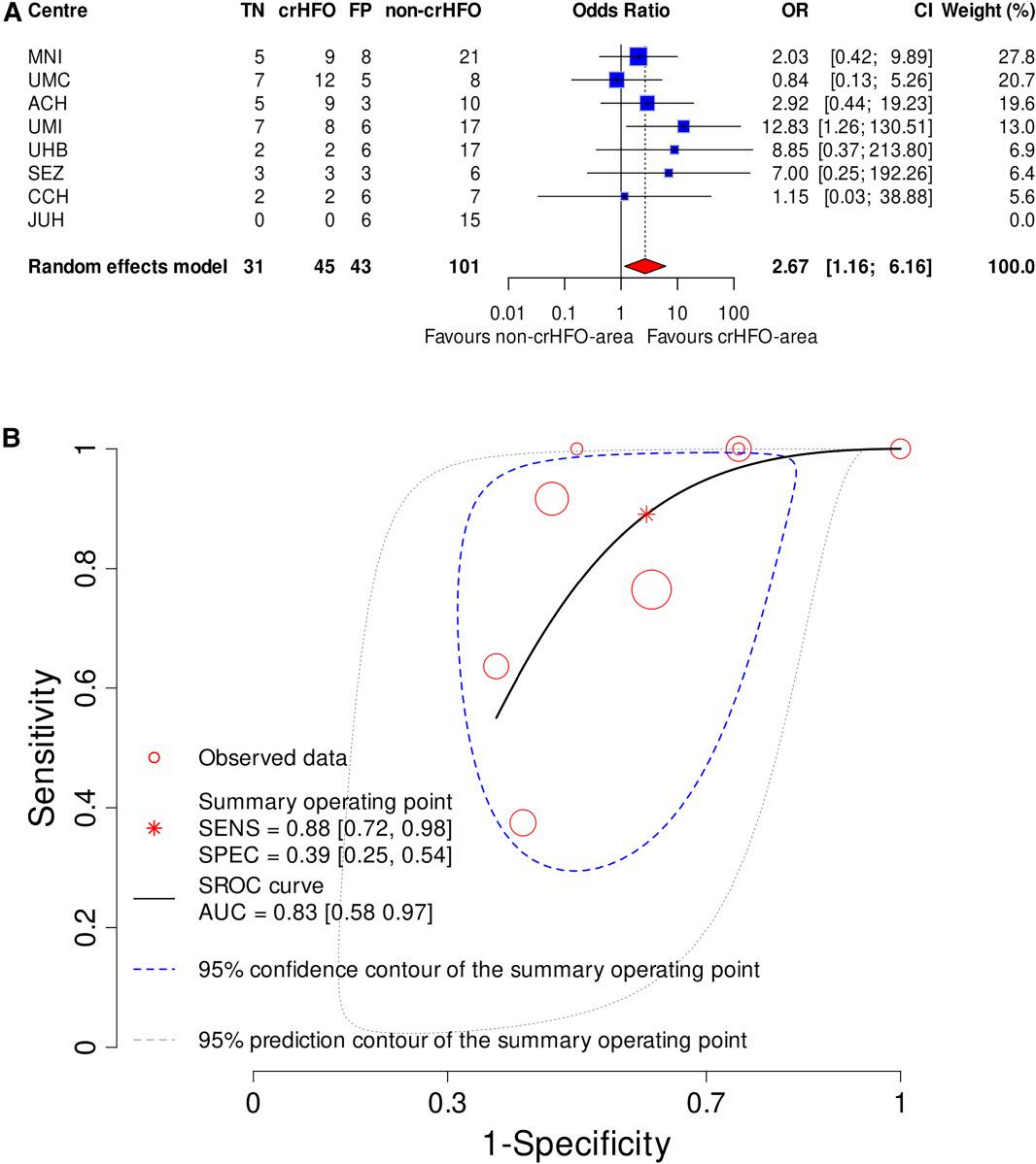
**HFO-area resection and seizure freedom.** (**A**) Association of crHFO-area and ILAE1. Each study centre is represented by a blue square. The size of the square represents the relative statistical weight of each study centre, with larger squares indicating study centres that carry greater statistical weight in the overall results. The pooled data with 95% CI is shown by a red diamond. The dotted vertical line represents the ‘line of null effect’. The likelihood of achieving ILAE1 was higher for crHFO-area compared with non-crHFO-area (OR = 2.67, CI 1.16–6.16, *P* = 0.02). (**B**) The predictive performance of crHFO-area for ILAE1. Red circles represent the study centres, where the size of the circle reflects the number of patients contributed by that centre. The model integrates the sensitivity and specificity across the centres and yields a summary receiver operating curve (SROC, black). The summary operating point is surrounded by the 95% confidence contour (blue). Note the wide spread between the centres, which might reflect the spread in iEEG recording set-ups and therapeutic strategies between centres. CI = confidence interval; crHFO-area = completely resected HFO area; HFO = high-frequency oscillation; ILAE = International League Against Epilepsy; iEEG = intracranial EEG; OR = odds ratio.

The likelihood of ILAE1 was higher for crHFO-area compared with non-crHFO-area (OR 2.67, CI 1.16–6.16, *P* = 0.02; Fig. 4A). The random effects model yielded low heterogeneity (*I*^2^ = 0%, *P* = 0.61) for the HFO area.

The pooled sensitivity and specificity were 0.88, CI 0.72–0.98 and 0.39, CI 0.25–0.54, respectively. The overall weighted AUC (Fig. 4B) for crHFO-area was 0.83, CI 0.58–0.97, indicating good predictive performance. Note the wide spread between the centres, which might reflect the spread in iEEG recording set-ups and therapeutic strategies between centres.

### Variables influencing the seizure outcome prediction

In addition to the analyses listed in the study protocol, we performed exploratory *post hoc* analyses. Initially, we compared the HFO rate between sEEG and ECoG channels within the HFO area. When comparing the 366 sEEG channels (median rate 4.0 HFO/min, interquartile range 2.3–7.9) with the 21 ECoG channels (median rate 2.1 HFO/min, interquartile range 1.3–6.8), we did not find a significant difference (*P* = 0.21, Wilcoxon rank-sum test). We then assessed which variables influenced the seizure outcome prediction using a linear mixed effects model (Table 1). Among the modelled mixed effects, the data variables that influenced the seizure outcome prediction were the electrode type and the complete resection of the HFO area. For patients with sEEG electrodes, the seizure outcome prediction was more likely to be correct than for patients with ECoG electrodes (*t*-value = 2.43, *P* = 0.016). As expected from our other statistical analyses, patients with complete resection of the HFO area were more likely to achieve seizure freedom (ILAE1, *t*-value = −2.98, *P* = 0.003).

**Table 1:** Predictors of seizure outcome

Effect	Median	Levels	Standard error	*t*-stat	*P*-value
Intercept	–	–	0.235	1.660	0.099
Age	29	0: [0–13]; 1: [14–18]; 2: [19–25]; 3: [26–59]; 4: [>60]; years	0.003	0.809	0.419
Sex	–	0: male; 1: female	0.080	−1.280	0.202
Pathology	–	0: not available; 1: non-FCD cortical lesion; 2: MTLE; 3: FCD	0.044	0.146	0.883
Electrode type	–	0: ECoG; 1: sEEG; 2: both	0.081	2.435	0.016*
Number of recording channels, *i*	78	0: [1–44]; 1: [45–104]; 2: [>105] channels	0.096	−0.404	0.686
Number of HFO area channels, *j*	2	0: 1; 1: [2–3]; 2: [4–7] channels	0.086	−0.697	0.486
Number of resected channels, *k*	11	0: [1–5]; 1: [6–24]; 2: [25–83] channels	0.059	0.713	0.476
crHFO-area	–	0: crHFO-area 1: non-crHFO-area	0.093	−2.985	0.003*
Follow-up duration	36.5	0: [24]; 1: [25–37]; 2: [38–280] months	0.026	0.377	0.706

Linear fixed effects model for seizure outcome prediction. crHFO-area = completely resected high-frequency oscillation area; FCD = focal cortical dysplasia; MTLE = mesial temporal lobe epilepsy.

*P < 0.05.

## Discussion

We present the results of a large multicentre observational study as an important milestone in HFO research. When considering the predictive value of crHFO-area and non-crHFO-area for ILAE1, we found an OR (2.67) that was significant but lower than in a recent meta-analysis (6.39),^11^ in which blinded data analysis was not performed in most studies. We believe our study advances the field in key aspects. First, this study represents the largest multicentre successful validation of prospective HFO analysis to date, involving eight independent epilepsy centres. We found several TP with a small number of channels in the HFO area (median of two channels per patient); in these patients, our HFO analysis would suggest a small planned RV if in agreement with other indices of epileptogenicity. Second, with its pre-registration,^9^ our study differs conceptually from several other recent studies that propose iEEG pattern analysis to guide epilepsy surgery.^22-24^ This ensures a higher degree of rigour and reproducibility in comparison to retrospective analyses or studies without pre-registration. Third, we used a fully automated HFO detection algorithm that was applied equally to datasets from other centres, using different equipment, without any additional training or adjustments. This approach enhances the generalizability of our findings across different recording set-ups, patient demographics and therapeutic strategies. The fully automated HFO detection was blinded to clinical information. This approach provides an unbiased evaluation of HFO resection across diverse centres with prospective data analysis.

As a limitation, the algorithm would not contribute to the clinical decision-making in the 9% of patients without conclusive HFO area. Conversely, this is a strong point of the algorithm because it clearly states its limits of applicability. As a further limitation, in FN patients the HFO area was completely resected but seizures recurred. This limitation could be from the inherent sparse sampling that limits all human iEEG: not all relevant HFOs might have been sampled by the electrode placement. Alternatively, other confounders, such as high background noise^25,26^ or high artefact load,^27^ could disrupt the algorithm, or there might be patients in whom HFOs are not completely representative of the EZ.

Another limitation is the low specificity (39%), because there were many FP patients who became seizure free without removal of the complete HFO-generating brain tissue. Removal of HFO-generating areas would have led to an extension of the RV in surgery without any benefit for the patient. Possibly, removing the most important hub in the HFO network might be sufficient to cure a patient.^28^ Alternatively, not all the HFO channels as defined by our detection pipeline were reliable markers of epileptogenic activity, or nearby resection could have disrupted the connectivity sufficiently. Our blinded multicentre analysis did not assess these complex possibilities. One might therefore consider the following pragmatic approach to use HFO data: if the original surgical plan does not include complete resection of the HFO area, it might be useful to reconsider the plan and see whether inclusion of the HFO area would make sense in the context of the particular situation of the patient (maybe the HFO area is part of the periphery of a lesion or there are other factors contributing to a decision to extend the resection). If the HFO area is not contiguous with the planned RV or shows no other indices of epileptogenicity, it might be better to ignore it. For example, among the 30 mesial temporal lobe epilepsy patients, six patients (Patients 114, 117, 120, 123, 124 and 125 in Supplementary Table 1) had an HFO area that extended beyond mesial temporal lobe epilepsy structures into neocortical regions. Note that in these patients, the HFO area is not contiguous, does not point to a single focus and was not completely resected.

It is also known that the resection is usually much larger than the region encompassing the contacts of the seizure onset zone. It is important to determine what else, other than the seizure onset zone, needs to be resected. The HFO findings in a patient should be interpreted considering the overall clinical picture. It might be that a new plan can be considered, including the HFO area but excluding non-HFO areas that were considered mildly epileptogenic but nevertheless included in the original plan; this alternative plan might not result in a larger resection but might increase the chances of seizure freedom given the potential epileptogenicity of the HFO area.

It is important to point out that we are not proposing HFOs as a method to supplant other clinical tools, nor to be used dogmatically to define resection margins. There are many complex decisions that go into the planned resection. Sometimes MRI or PET lesions are not fully resected, sometimes all the spiking channels or even the entire seizure onset zone is not fully resected; this is standard of care. Clinicians often acquire a new study modality, e.g. magnetoencephalography or PET, to help define the extent of the epileptic network. Such extra information is not considered dogma but is greatly appreciated by the clinical team. HFOs can serve that same purpose.

As an outlook, it seems promising to investigate iEEG patterns that combine HFO analysis with other features of the iEEG.^22-24,29-33^ However, the validity of these patterns still needs to be tested in a multicentre study protocol with prospective data analysis.

## Conclusion

In conclusion, the fully automated analysis of real-life clinical data encourages implementation of the method in a push-button commercial software package, a prerequisite for its integration in clinical practice. The results of our HFO analysis might be helpful if adaptations to a surgical plan are considered.

## Supplementary Material

awae361_Supplementary_Data

## Data Availability

All data needed to evaluate the conclusions in the article are present in the article. The code is indexed at https://HFOzuri.ch/. The raw iEEG data cannot be published open access given restrictions imposed by the various local ethics committees.
