# Supplementary material for: Multicentre analysis of seizure outcome predicted by removal of high-frequency oscillations
Source: Brain. 2024 Nov 12;148(5):1769–77. doi: 10.1093/brain/awae361 (PMC12073983; doi:10.1093/brain/awae361)

Supplementary Figure 1:

Patient example how sEEG channels are labelled to "HFO area"

Overview of the HFO rate distribution per 5-min NREM-sleep epoch (red vertical bar) of an individual patient (MNI_0953, adult male with FCD IIa, 79 bipolar sEEG channels, 12 NREM-sleep epochs of 5 min each). The channels with rates exceeding the 95th percentile (black line indicating the HFO rate threshold, set at 1.8 HFOs/min) were considered high-rate channels. Since their HFO rate was consistently high for ≥50% of the available epochs, these channels defined a conclusive HFO area (red channels). In this example patient, the HFO area comprised bi-polar channels RLI01-RLI02, RLI02-RLI03, RLI03-RLI04 that were completely resected. The patient achieved postoperative seizure freedom (follow-up = 126 months) and was labelled true negative (TN).

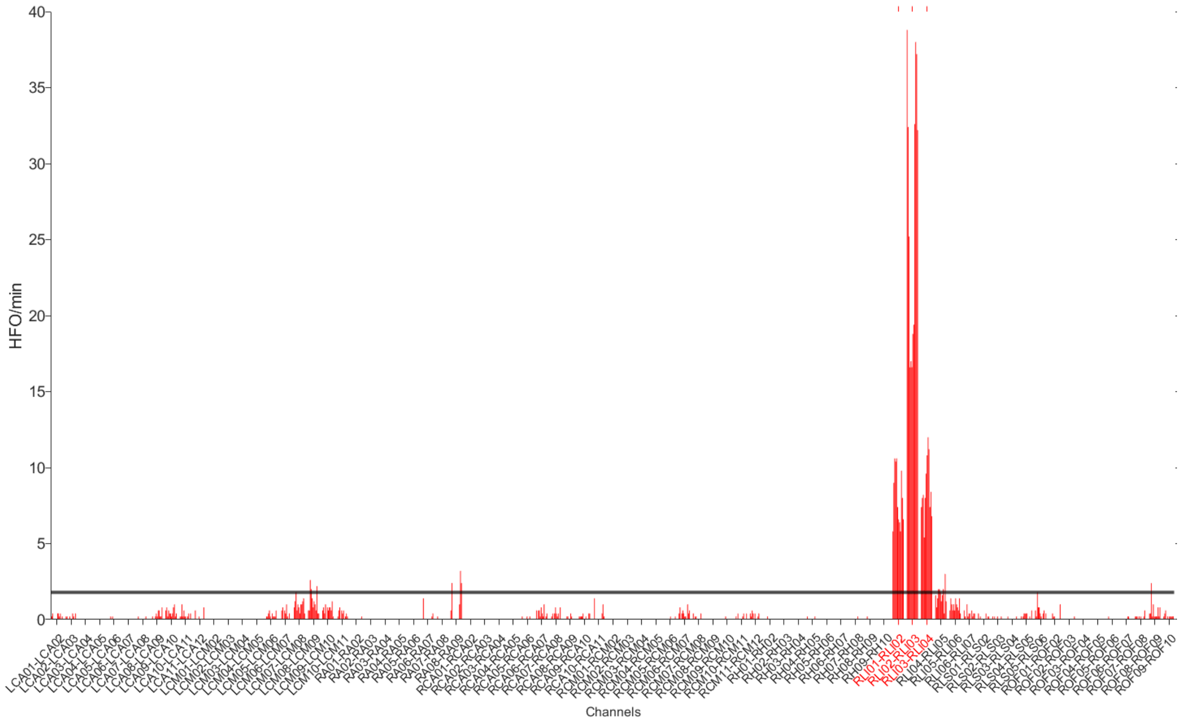

Supplement: awae361_Supplementary_Data [file awae361_supplementary_data.zip › brain-2024-01403-File008.pdf]
